# The Japanese View Of “Successful Aging”: The Impact Of Family Norms On The Desire to Live Long

**DOI:** 10.1093/geroni/igaf122.1122

**Published:** 2025-12-31

**Authors:** Saori Yasumoto, Junya Tsutsui, Keiko Tanaka

**Affiliations:** Osaka University, Osaka, Osaka, Japan; Ritsumeikan University, Kyoto, Kyoto, Japan; Meiji Gakuin University, MInato, Tokyo, Japan

## Abstract

We aimed to understand how people’s beliefs and expectations about family norms influence their desire to live long in Japan. Japan is the world’s leading super-aged society, with the highest ratio of centenarians per 10,000 people; however, researchers report that fewer older people wish to live longer than those who do not. The most common reason for unwillingness to live long is the perceived burdensomeness of old age on their family. Such negative views on aging affect their physical and psychological health; therefore, the factors for such thoughts should be identified to reconsider it. The data used for the analysis came from 2475 people aged between 50 and 79 living in Japan and were derived from a nationwide random sample survey conducted in 2024. We used ordered logistic regression and tried to find a model that fits the data by applying family norms as an explanatory variable and other demographic variables (e.g., gender and marital status). People with a strong desire for longevity tended to have conservative views regarding family norms (e.g., younger family members should take care of older family members). Successful aging has often been defined based on the medical model, which focuses on health and independence (Rowe & Khan, 1997), and the psychological model, which emphasizes the psychological adaptation to losses in aging (Baltes & Baltes, 1990). Our findings introduce a new model of successful aging by demonstrating how culture as a social structure, especially beliefs in family norms, affects how we perceive aging.

